# Supplementary material for: Rosiglitazone Does Not Show Major Hidden Cardiotoxicity in Models of Ischemia/Reperfusion but Abolishes Ischemic Preconditioning-Induced Antiarrhythmic Effects in Rats In Vivo
Source: Pharmaceuticals (Basel). 2022 Aug 26;15(9):1055. doi: 10.3390/ph15091055 (PMC9503202; doi:10.3390/ph15091055)
Supplement: Supplementary file 1 [file pharmaceuticals-15-01055-s001.zip › pharmaceuticals-1815315-SI.pdf]

Supplementary Figure S1.

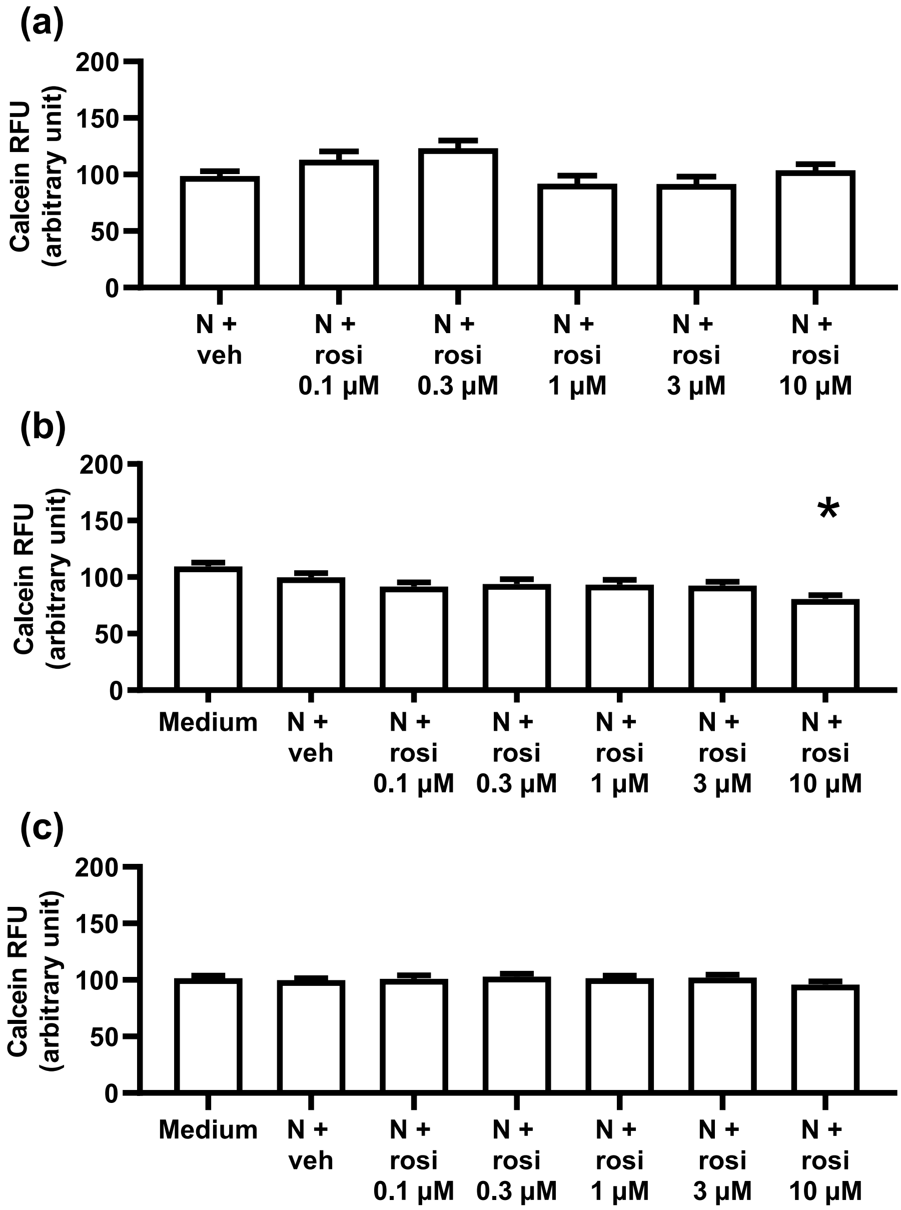

Supplementary Figure S1. Cell viability in normoxic conditions with or without varying rosiglitazone concentrations. Results are presented as mean with standard error. **(a): Adult rat cardiomyocytes.** Normoxia (N) + vehicle group was set to 100% relative fluorescent units (RFU), and all of the data were normalized to the averaged N + vehicle group. Rosiglitazone treatment did not aggravate cell death in normoxic conditions in adult rat cardiomyocytes. (Kruskal–Wallis, Dunn´s post hoc, * $p < 0.05$ vs. N + vehicle, n=7-9, 25-66 technical replicates) **(b): AC16 cell line.** Normoxia (N) + vehicle group was set to 100% RFU, and all of the data were normalized to the averaged medium treated group. Rosiglitazone treatment aggravated cell death in normoxic conditions in AC16 cells with 10 µM. (Kruskal–Wallis, Dunn´s post hoc, * $p < 0.05$ vs. N + vehicle, n=6-7, 69-108 technical replicates) **(c): Differentiated AC16 cell line.** Normoxia (N) + vehicle group was set to 100% RFU, and all of the data were normalized to the averaged medium treated group. Rosiglitazone treatment did not aggravate cell death in normoxic conditions in differentiated AC16 cells. (Kruskal–Wallis, Dunn´s post hoc, * $p < 0.05$ vs. N + vehicle. n=5, 31-73 technical replicates)

N: normoxia. **veh:** vehicle. **rosi:** rosiglitazone. **RFU:** relative flourescence unit.

Supplementary Table S1.

Body-weight-development of rats under chronic rosiglitazone treatment

	day-1	day0	day1	day2	day3	day4	day5	day6	day7	day8
Mean +/- SEM vehicle	96.1 ±2.3	102.5 ±1.9	111.1 ±2.0	118.9 ±2.1	125.9 ±2.2	134.9 ±2.3	143.0 ±2.4	150.7 ±2.5	158.4 ±2.5	166.8 ±2.6
Mean +/- SEM rosiglitazone	94.7 ±2.3	103.2 ±1.7	110.7 ±1.8	118.5 ±1.9	125.9 ±2.0	133.9 ±2.0	141.5 ±2.2	148.1 ±2.1	156.1 ±2.2	163.4 ±2.3

	day9	day10	day11	day12	day13	day14	day15	day16	day17	day18
Mean +/- SEM vehicle	174.3 ±2.7	180.3 ±3.5	190.1 ±3.0	197.0 ±3.0	204.1 ±3.0	211.5 ±3.2	221.8 ±4.0	226.5 ±3.4	234.1 ±3.5	242.4 ±3.6
Mean +/- SEM rosiglitazone	170.5 ±2.4	177.5 ±2.4	185.5 ±2.7	192.1 ±2.7	198.6 ±2.8	204.8 ±2.8	212.3 ±2.8	219.2 ±3.0	225.9 ±2.9	234.3 ±3.0

	day19	day20	day21	day22	day23	day24	day25	day26	day27	day28
Mean +/- SEM vehicle	249.7 ±3.6	257.0 ±3.7	263.7 ±4.0	272.3 ±3.8	277.9 ±4.1	284.3 ±4.3	290.6 ±4.5	293.7 ±5.4	302.6 ±4.8	307.8 ±4.8
Mean +/- SEM rosiglitazone	241.4 ±3.1	245.0 ±3.8	254.2 ±3.2	261.3 ±3.3	267.9 ±3.4	271.5 ±3.7	278.7 ±3.4	282.4 ±4.5	288.9 ±3.8	295.6 ±3.8

Supplementary Table S1. Weight development of male Wistar rats under chronic rosiglitazone treatment vs. vehicle. Results are presented as mean ± SEM. During the 28-day treatment, the weight gain did not change with rosiglitazone treatment compared to the vehicle group (1% hydroxyethyl-cellulose).(Mixed effect analysis, Sidak´s post hoc, day -1: n=25, day 0-28: n=40)

Supplementary Table S2.

Heartratemeasurementduringsurgicalprotocolinrats

surgical period	start	ischemia		reperfusion				
time	0 min	45 min	60 min	75 min	100 min	130 min	160 min	190 min
Mean +/- SEM<br>I/R vehicle	452<br>±15	429<br>±19	386<br>±41	416<br>±17	412<br>±19	430*<br>±16	431<br>±21	417<br>±22
Mean +/- SEM<br>I/R rosiglitazone	427<br>±11	436<br>±23	426<br>±20	426*<br>±12	425<br>±15	402<br>±11	396<br>±18	410<br>±17
Mean +/- SEM<br>IPC vehicle	425<br>±18	422<br>±18	387<br>±14	395<br>±16	401<br>±14	396<br>±14	404<br>±14	404<br>±18
Mean +/- SEM<br>IPC rosiglitazone	427<br>±30	397<br>±13	390<br>±21	375<br>±13	378<br>±15	368<br>±15	369<br>±15	367<br>±16

Supplementary Table S2. Mean heart rate was monitored throughout the entire in vivo surgery protocol. Results are presented as mean ± SEM. There is no meaningful difference between the treatment groups with respect to the heart rate. (Mixed effect analysis, Tukey´s post hoc, * $p < 0.05$ vs. IPC rosiglitazone, n=9-13)

Supplementary Table S3.

Blood pressure measurement during surgical protocol in rats

Surgical period	start	ischemia		reperfusion				
time	0 min	45 min	60 min	75 min	100 min	130 min	160 min	190 min
Mean +/- SEM I/R vehicle	96,3 ±9	81,1 ±6,2	60,9 ±7	78,1 ±6,9	75,3 ±4,1	78,8 ±4,7	75,4 ±5,4	76,5 ±4,5
Mean +/- SEM I/R rosiglitazone	102,2 ±8,9	63,2 ±8,6	76 ±8,2	85,3 ±12,7	85,4 ±6,7	83,2 ±7,1	91,7 ±8,5	86,7 ±6,8
Mean +/- SEM IPC vehicle	96,2 ±7	74,5 ±5,3	62,7 ±6,6	89,6 ±3,9	84,2 ±4,8	80,6 ±5,4	79,9 ±3	80,5 ±5,8
Mean +/- SEM IPC rosiglitazone	93,8 ±4,7	75,2 ±5,1	68 ±6,1	78,3 ±6,1	80,3 ±5,3	74 ±5,6	77,2 ±4,4	75,4 ±5,6

Supplementary Table S3. Mean arterial blood pressure was monitored throughout the entire in vivo surgery protocol. Results are presented as mean ± SEM. There is no significant difference between the treatment groups with respect to the blood pressure. (Mixed effect analysis, Tukey´s post hoc, n=9-13)
